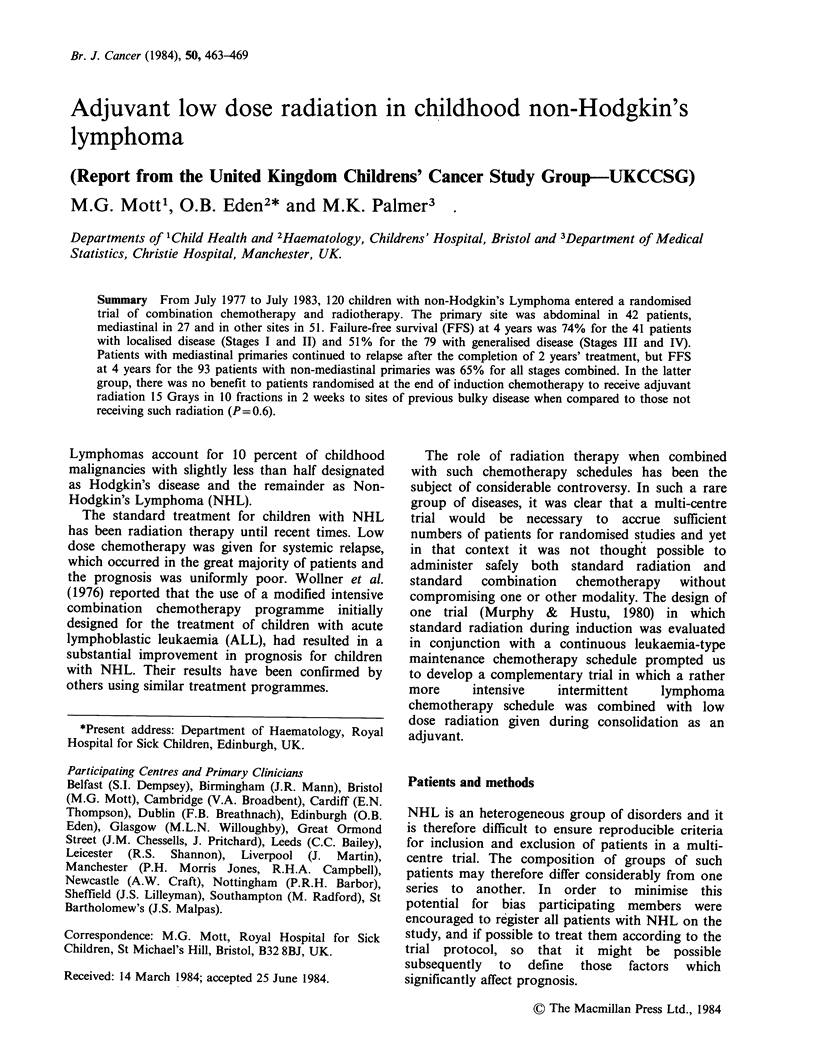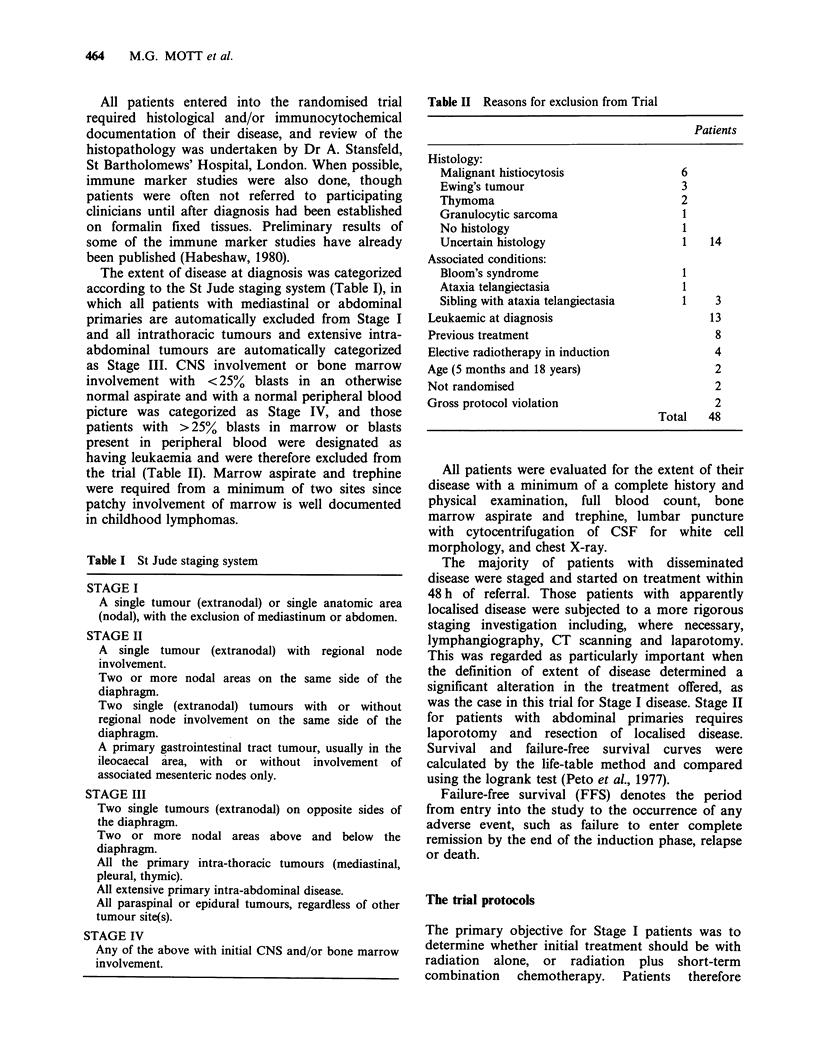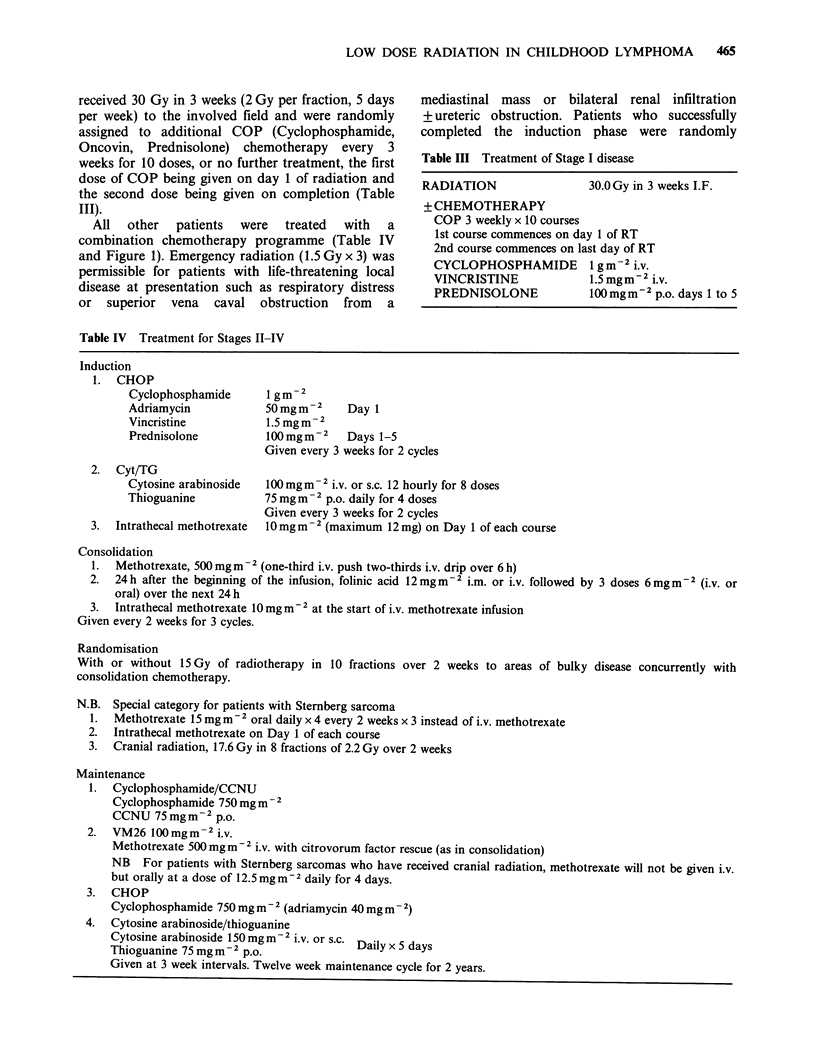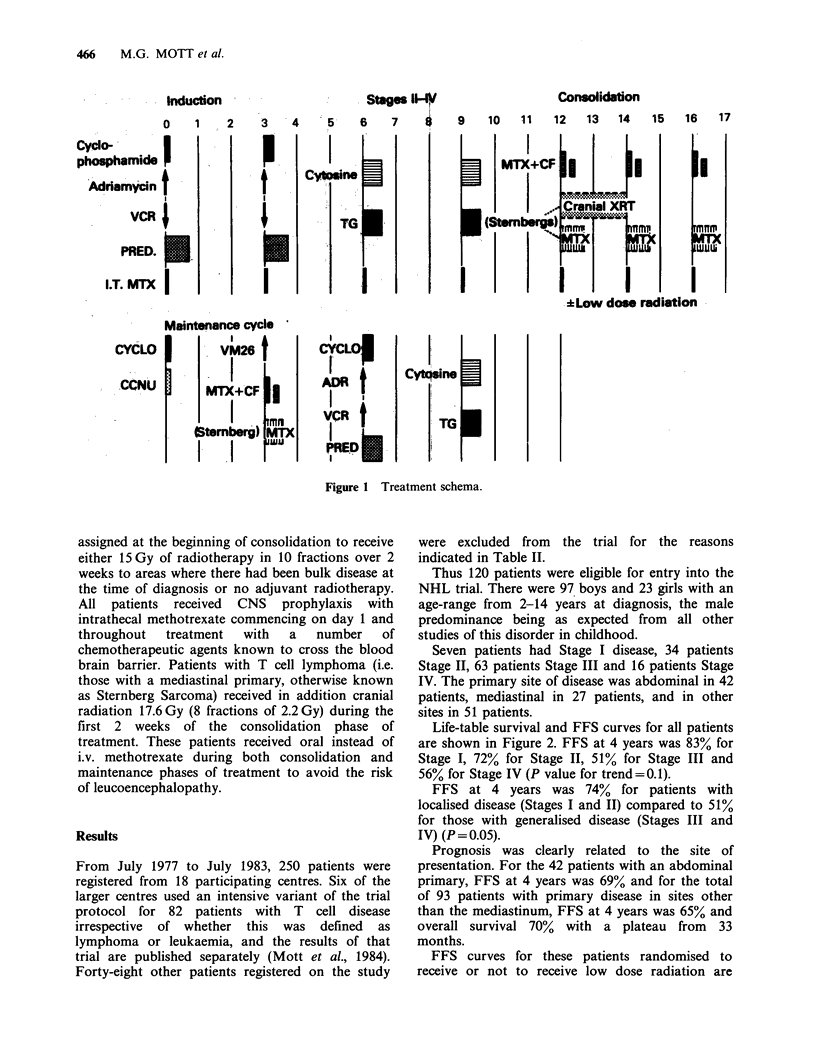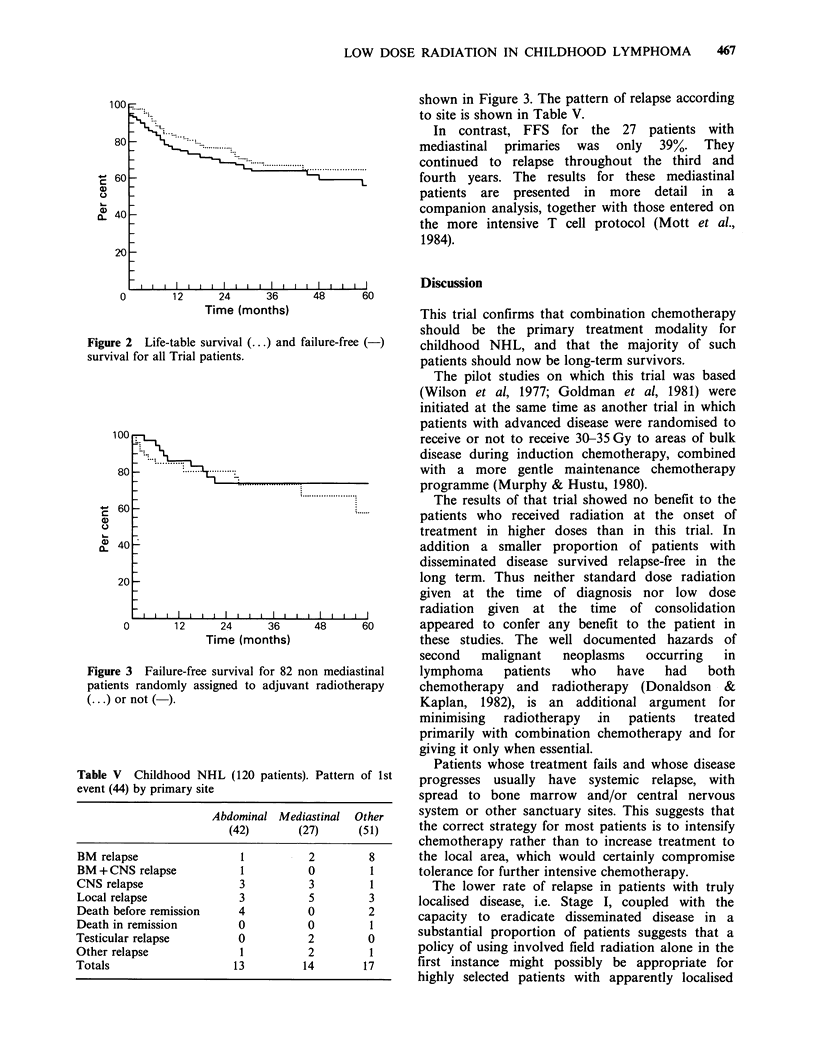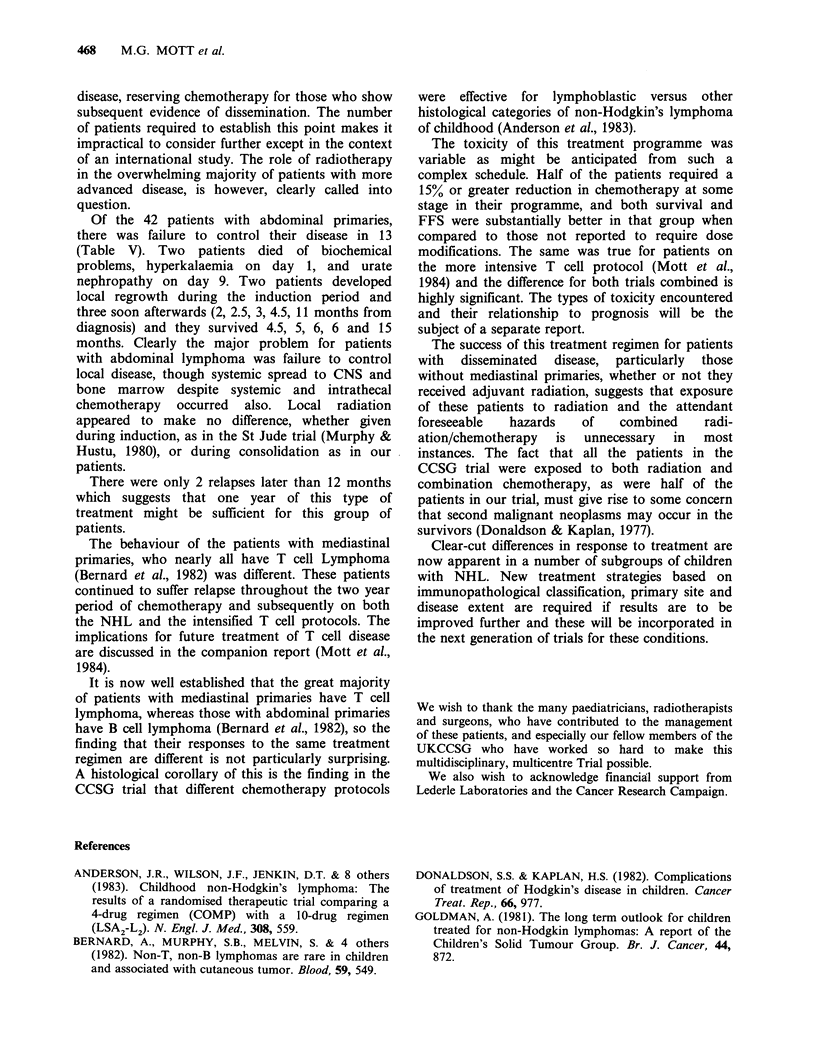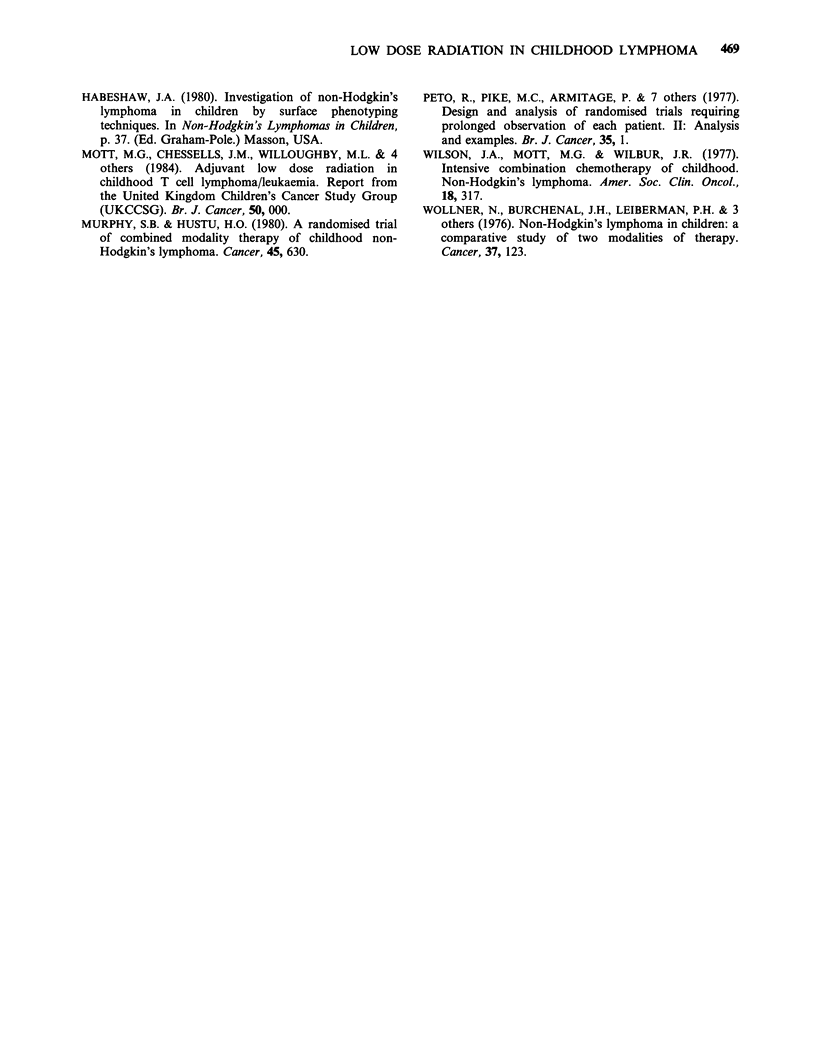# Adjuvant low dose radiation in childhood non-Hodgkin's lymphoma (report from the United Kingdom Childrens' Cancer Study Group--UKCCSG).

**DOI:** 10.1038/bjc.1984.202

**Published:** 1984-10

**Authors:** M. G. Mott, O. B. Eden, M. K. Palmer

## Abstract

From July 1977 to July 1983, 120 children with non-Hodgkin's Lymphoma entered a randomised trial of combination chemotherapy and radiotherapy. The primary site was abdominal in 42 patients, mediastinal in 27 and in other sites in 51. Failure-free survival (FFS) at 4 years was 74% for the 41 patients with localised disease (Stages I and II) and 51% for the 79 with generalised disease (Stages III and IV). Patients with mediastinal primaries continued to relapse after the completion of 2 years' treatment, but FFS at 4 years for the 93 patients with non-mediastinal primaries was 65% for all stages combined. In the latter group, there was no benefit to patients randomised at the end of induction chemotherapy to receive adjuvant radiation 15 Grays in 10 fractions in 2 weeks to sites of previous bulky disease when compared to those not receiving such radiation (P = 0.6).


*Br. J. Cancer* (1984), **50**, 463–469

### Adjuvant low dose radiation in childhood non-Hodgkin's lymphoma

### (Report from the United Kingdom Childrens' Cancer Study Group—UKCCSG)

M.G. Mott[1], O.B. Eden[2*] and M.K. Palmer[3]

*Departments of [1]Child Health and [2]Haematology, Childrens' Hospital, Bristol and [3]Department of Medical Statistics, Christie Hospital, Manchester, UK.*

**Summary** From July 1977 to July 1983, 120 children with non-Hodgkin's Lymphoma entered a randomised trial of combination chemotherapy and radiotherapy. The primary site was abdominal in 42 patients, mediastinal in 27 and in other sites in 51. Failure-free survival (FFS) at 4 years was 74% for the 41 patients with localised disease (Stages I and II) and 51% for the 79 with generalised disease (Stages III and IV). Patients with mediastinal primaries continued to relapse after the completion of 2 years' treatment, but FFS at 4 years for the 93 patients with non-mediastinal primaries was 65% for all stages combined. In the latter group, there was no benefit to patients randomised at the end of induction chemotherapy to receive adjuvant radiation 15 Grays in 10 fractions in 2 weeks to sites of previous bulky disease when compared to those not receiving such radiation ($P=0.6$).

Lymphomas account for 10 percent of childhood malignancies with slightly less than half designated as Hodgkin's disease and the remainder as Non-Hodgkin's Lymphoma (NHL).

The standard treatment for children with NHL has been radiation therapy until recent times. Low dose chemotherapy was given for systemic relapse, which occurred in the great majority of patients and the prognosis was uniformly poor. Wollner *et al.* (1976) reported that the use of a modified intensive combination chemotherapy programme initially designed for the treatment of children with acute lymphoblastic leukaemia (ALL), had resulted in a substantial improvement in prognosis for children with NHL. Their results have been confirmed by others using similar treatment programmes.

*Present address: Department of Haematology, Royal Hospital for Sick Children, Edinburgh, UK.

*Participating Centres and Primary Clinicians*
Belfast (S.I. Dempsey), Birmingham (J.R. Mann), Bristol (M.G. Mott), Cambridge (V.A. Broadbent), Cardiff (E.N. Thompson), Dublin (F.B. Breathnach), Edinburgh (O.B. Eden), Glasgow (M.L.N. Willoughby), Great Ormond Street (J.M. Chessells, J. Pritchard), Leeds (C.C. Bailey), Leicester (R.S. Shannon), Liverpool (J. Martin), Manchester (P.H. Morris Jones, R.H.A. Campbell), Newcastle (A.W. Craft), Nottingham (P.R.H. Barbor), Sheffield (J.S. Lilleyman), Southampton (M. Radford), St Bartholomew's (J.S. Malpas).

Correspondence: M.G. Mott, Royal Hospital for Sick Children, St Michael's Hill, Bristol, B32 8BJ, UK.

Received: 14 March 1984; accepted 25 June 1984.

The role of radiation therapy when combined with such chemotherapy schedules has been the subject of considerable controversy. In such a rare group of diseases, it was clear that a multi-centre trial would be necessary to accrue sufficient numbers of patients for randomised studies and yet in that context it was not thought possible to administer safely both standard radiation and standard combination chemotherapy without compromising one or other modality. The design of one trial (Murphy & Hustu, 1980) in which standard radiation during induction was evaluated in conjunction with a continuous leukaemia-type maintenance chemotherapy schedule prompted us to develop a complementary trial in which a rather more intensive intermittent lymphoma chemotherapy schedule was combined with low dose radiation given during consolidation as an adjuvant.

### Patients and methods

NHL is an heterogeneous group of disorders and it is therefore difficult to ensure reproducible criteria for inclusion and exclusion of patients in a multi-centre trial. The composition of groups of such patients may therefore differ considerably from one series to another. In order to minimise this potential for bias participating members were encouraged to register all patients with NHL on the study, and if possible to treat them according to the trial protocol, so that it might be possible subsequently to define those factors which significantly affect prognosis.

© The Macmillan Press Ltd., 1984

All patients entered into the randomised trial required histological and/or immunocytochemical documentation of their disease, and review of the histopathology was undertaken by Dr A. Stansfeld, St Bartholomews' Hospital, London. When possible, immune marker studies were also done, though patients were often not referred to participating clinicians until after diagnosis had been established on formalin fixed tissues. Preliminary results of some of the immune marker studies have already been published (Habeshaw, 1980).

The extent of disease at diagnosis was categorized according to the St Jude staging system (Table I), in which all patients with mediastinal or abdominal primaries are automatically excluded from Stage I and all intrathoracic tumours and extensive intra-abdominal tumours are automatically categorized as Stage III. CNS involvement or bone marrow involvement with <25% blasts in an otherwise normal aspirate and with a normal peripheral blood picture was categorized as Stage IV, and those patients with >25% blasts in marrow or blasts present in peripheral blood were designated as having leukaemia and were therefore excluded from the trial (Table II). Marrow aspirate and trephine were required from a minimum of two sites since patchy involvement of marrow is well documented in childhood lymphomas.

**Table I** St Jude staging system

STAGE I
- A single tumour (extranodal) or single anatomic area (nodal), with the exclusion of mediastinum or abdomen.

STAGE II
- A single tumour (extranodal) with regional node involvement.
- Two or more nodal areas on the same side of the diaphragm.
- Two single (extranodal) tumours with or without regional node involvement on the same side of the diaphragm.
- A primary gastrointestinal tract tumour, usually in the ileocaecal area, with or without involvement of associated mesenteric nodes only.

STAGE III
- Two single tumours (extranodal) on opposite sides of the diaphragm.
- Two or more nodal areas above and below the diaphragm.
- All the primary intra-thoracic tumours (mediastinal, pleural, thymic).
- All extensive primary intra-abdominal disease.
- All paraspinal or epidural tumours, regardless of other tumour site(s).

STAGE IV
- Any of the above with initial CNS and/or bone marrow involvement.

**Table II** Reasons for exclusion from Trial

| | | *Patients* |
|---|---|---|
| Histology: | | |
| Malignant histiocytosis | 6 | |
| Ewing's tumour | 3 | |
| Thymoma | 2 | |
| Granulocytic sarcoma | 1 | |
| No histology | 1 | |
| Uncertain histology | 1 | 14 |
| Associated conditions: | | |
| Bloom's syndrome | 1 | |
| Ataxia telangiectasia | 1 | |
| Sibling with ataxia telangiectasia | 1 | 3 |
| Leukaemic at diagnosis | | 13 |
| Previous treatment | | 8 |
| Elective radiotherapy in induction | | 4 |
| Age (5 months and 18 years) | | 2 |
| Not randomised | | 2 |
| Gross protocol violation | | 2 |
| | Total | 48 |

All patients were evaluated for the extent of their disease with a minimum of a complete history and physical examination, full blood count, bone marrow aspirate and trephine, lumbar puncture with cytocentrifugation of CSF for white cell morphology, and chest X-ray.

The majority of patients with disseminated disease were staged and started on treatment within 48 h of referral. Those patients with apparently localised disease were subjected to a more rigorous staging investigation including, where necessary, lymphangiography, CT scanning and laparotomy. This was regarded as particularly important when the definition of extent of disease determined a significant alteration in the treatment offered, as was the case in this trial for Stage I disease. Stage II for patients with abdominal primaries requires laporotomy and resection of localised disease. Survival and failure-free survival curves were calculated by the life-table method and compared using the logrank test (Peto *et al.*, 1977).

Failure-free survival (FFS) denotes the period from entry into the study to the occurrence of any adverse event, such as failure to enter complete remission by the end of the induction phase, relapse or death.

### The trial protocols

The primary objective for Stage I patients was to determine whether initial treatment should be with radiation alone, or radiation plus short-term combination chemotherapy. Patients therefore received 30 Gy in 3 weeks (2 Gy per fraction, 5 days per week) to the involved field and were randomly assigned to additional COP (Cyclophosphamide, Oncovin, Prednisolone) chemotherapy every 3 weeks for 10 doses, or no further treatment, the first dose of COP being given on day 1 of radiation and the second dose being given on completion (Table III).

All other patients were treated with a combination chemotherapy programme (Table IV and Figure 1). Emergency radiation (1.5 Gy × 3) was permissible for patients with life-threatening local disease at presentation such as respiratory distress or superior vena caval obstruction from a mediastinal mass or bilateral renal infiltration ± ureteric obstruction. Patients who successfully completed the induction phase were randomly

**Table III** Treatment of Stage I disease

| | |
|---|---|
| RADIATION | 30.0 Gy in 3 weeks I.F. |
| ± CHEMOTHERAPY | |
| COP 3 weekly × 10 courses | |
| 1st course commences on day 1 of RT | |
| 2nd course commences on last day of RT | |
| CYCLOPHOSPHAMIDE | 1 g m$^{-2}$ i.v. |
| VINCRISTINE | 1.5 mg m$^{-2}$ i.v. |
| PREDNISOLONE | 100 mg m$^{-2}$ p.o. days 1 to 5 |

**Table IV** Treatment for Stages II–IV

Induction

1. CHOP

| | | |
|---|---|---|
| Cyclophosphamide | 1 g m$^{-2}$ | |
| Adriamycin | 50 mg m$^{-2}$ | Day 1 |
| Vincristine | 1.5 mg m$^{-2}$ | |
| Prednisolone | 100 mg m$^{-2}$ | Days 1–5 |
| | Given every 3 weeks for 2 cycles | |

2. Cyt/TG

| | |
|---|---|
| Cytosine arabinoside | 100 mg m$^{-2}$ i.v. or s.c. 12 hourly for 8 doses |
| Thioguanine | 75 mg m$^{-2}$ p.o. daily for 4 doses |
| | Given every 3 weeks for 2 cycles |

3. Intrathecal methotrexate 10 mg m$^{-2}$ (maximum 12 mg) on Day 1 of each course

Consolidation

1. Methotrexate, 500 mg m$^{-2}$ (one-third i.v. push two-thirds i.v. drip over 6 h)
2. 24 h after the beginning of the infusion, folinic acid 12 mg m$^{-2}$ i.m. or i.v. followed by 3 doses 6 mg m$^{-2}$ (i.v. or oral) over the next 24 h
3. Intrathecal methotrexate 10 mg m$^{-2}$ at the start of i.v. methotrexate infusion

Given every 2 weeks for 3 cycles.

Randomisation

With or without 15 Gy of radiotherapy in 10 fractions over 2 weeks to areas of bulky disease concurrently with consolidation chemotherapy.

N.B. Special category for patients with Sternberg sarcoma

1. Methotrexate 15 mg m$^{-2}$ oral daily × 4 every 2 weeks × 3 instead of i.v. methotrexate
2. Intrathecal methotrexate on Day 1 of each course
3. Cranial radiation, 17.6 Gy in 8 fractions of 2.2 Gy over 2 weeks

Maintenance

1. Cyclophosphamide/CCNU
   Cyclophosphamide 750 mg m$^{-2}$
   CCNU 75 mg m$^{-2}$ p.o.
2. VM26 100 mg m$^{-2}$ i.v.
   Methotrexate 500 mg m$^{-2}$ i.v. with citrovorum factor rescue (as in consolidation)
   NB For patients with Sternberg sarcomas who have received cranial radiation, methotrexate will not be given i.v. but orally at a dose of 12.5 mg m$^{-2}$ daily for 4 days.
3. CHOP
   Cyclophosphamide 750 mg m$^{-2}$ (adriamycin 40 mg m$^{-2}$)
4. Cytosine arabinoside/thioguanine
   Cytosine arabinoside 150 mg m$^{-2}$ i.v. or s.c. Daily × 5 days
   Thioguanine 75 mg m$^{-2}$ p.o.
   Given at 3 week intervals. Twelve week maintenance cycle for 2 years.

**Figure 1** Treatment schema.

assigned at the beginning of consolidation to receive either 15 Gy of radiotherapy in 10 fractions over 2 weeks to areas where there had been bulk disease at the time of diagnosis or no adjuvant radiotherapy. All patients received CNS prophylaxis with intrathecal methotrexate commencing on day 1 and throughout treatment with a number of chemotherapeutic agents known to cross the blood brain barrier. Patients with T cell lymphoma (i.e. those with a mediastinal primary, otherwise known as Sternberg Sarcoma) received in addition cranial radiation 17.6 Gy (8 fractions of 2.2 Gy) during the first 2 weeks of the consolidation phase of treatment. These patients received oral instead of i.v. methotrexate during both consolidation and maintenance phases of treatment to avoid the risk of leucoencephalopathy.

### Results

From July 1977 to July 1983, 250 patients were registered from 18 participating centres. Six of the larger centres used an intensive variant of the trial protocol for 82 patients with T cell disease irrespective of whether this was defined as lymphoma or leukaemia, and the results of that trial are published separately (Mott *et al.*, 1984). Forty-eight other patients registered on the study were excluded from the trial for the reasons indicated in Table II.

Thus 120 patients were eligible for entry into the NHL trial. There were 97 boys and 23 girls with an age-range from 2–14 years at diagnosis, the male predominance being as expected from all other studies of this disorder in childhood.

Seven patients had Stage I disease, 34 patients Stage II, 63 patients Stage III and 16 patients Stage IV. The primary site of disease was abdominal in 42 patients, mediastinal in 27 patients, and in other sites in 51 patients.

Life-table survival and FFS curves for all patients are shown in Figure 2. FFS at 4 years was 83% for Stage I, 72% for Stage II, 51% for Stage III and 56% for Stage IV ($P$ value for trend = 0.1).

FFS at 4 years was 74% for patients with localised disease (Stages I and II) compared to 51% for those with generalised disease (Stages III and IV) ($P = 0.05$).

Prognosis was clearly related to the site of presentation. For the 42 patients with an abdominal primary, FFS at 4 years was 69% and for the total of 93 patients with primary disease in sites other than the mediastinum, FFS at 4 years was 65% and overall survival 70% with a plateau from 33 months.

FFS curves for these patients randomised to receive or not to receive low dose radiation are

**Figure 2** Life-table survival (...) and failure-free (—) survival for all Trial patients.

**Figure 3** Failure-free survival for 82 non mediastinal patients randomly assigned to adjuvant radiotherapy (...) or not (—).

**Table V** Childhood NHL (120 patients). Pattern of 1st event (44) by primary site

| | *Abdominal* (42) | *Mediastinal* (27) | *Other* (51) |
|---|---|---|---|
| BM relapse | 1 | 2 | 8 |
| BM + CNS relapse | 1 | 0 | 1 |
| CNS relapse | 3 | 3 | 1 |
| Local relapse | 3 | 5 | 3 |
| Death before remission | 4 | 0 | 2 |
| Death in remission | 0 | 0 | 1 |
| Testicular relapse | 0 | 2 | 0 |
| Other relapse | 1 | 2 | 1 |
| Totals | 13 | 14 | 17 |

shown in Figure 3. The pattern of relapse according to site is shown in Table V.

In contrast, FFS for the 27 patients with mediastinal primaries was only 39%. They continued to relapse throughout the third and fourth years. The results for these mediastinal patients are presented in more detail in a companion analysis, together with those entered on the more intensive T cell protocol (Mott *et al.*, 1984).

### Discussion

This trial confirms that combination chemotherapy should be the primary treatment modality for childhood NHL, and that the majority of such patients should now be long-term survivors.

The pilot studies on which this trial was based (Wilson *et al*, 1977; Goldman *et al*, 1981) were initiated at the same time as another trial in which patients with advanced disease were randomised to receive or not to receive 30–35 Gy to areas of bulk disease during induction chemotherapy, combined with a more gentle maintenance chemotherapy programme (Murphy & Hustu, 1980).

The results of that trial showed no benefit to the patients who received radiation at the onset of treatment in higher doses than in this trial. In addition a smaller proportion of patients with disseminated disease survived relapse-free in the long term. Thus neither standard dose radiation given at the time of diagnosis nor low dose radiation given at the time of consolidation appeared to confer any benefit to the patient in these studies. The well documented hazards of second malignant neoplasms occurring in lymphoma patients who have had both chemotherapy and radiotherapy (Donaldson & Kaplan, 1982), is an additional argument for minimising radiotherapy in patients treated primarily with combination chemotherapy and for giving it only when essential.

Patients whose treatment fails and whose disease progresses usually have systemic relapse, with spread to bone marrow and/or central nervous system or other sanctuary sites. This suggests that the correct strategy for most patients is to intensify chemotherapy rather than to increase treatment to the local area, which would certainly compromise tolerance for further intensive chemotherapy.

The lower rate of relapse in patients with truly localised disease, i.e. Stage I, coupled with the capacity to eradicate disseminated disease in a substantial proportion of patients suggests that a policy of using involved field radiation alone in the first instance might possibly be appropriate for highly selected patients with apparently localised disease, reserving chemotherapy for those who show subsequent evidence of dissemination. The number of patients required to establish this point makes it impractical to consider further except in the context of an international study. The role of radiotherapy in the overwhelming majority of patients with more advanced disease, is however, clearly called into question.

Of the 42 patients with abdominal primaries, there was failure to control their disease in 13 (Table V). Two patients died of biochemical problems, hyperkalaemia on day 1, and urate nephropathy on day 9. Two patients developed local regrowth during the induction period and three soon afterwards (2, 2.5, 3, 4.5, 11 months from diagnosis) and they survived 4.5, 5, 6, 6 and 15 months. Clearly the major problem for patients with abdominal lymphoma was failure to control local disease, though systemic spread to CNS and bone marrow despite systemic and intrathecal chemotherapy occurred also. Local radiation appeared to make no difference, whether given during induction, as in the St Jude trial (Murphy & Hustu, 1980), or during consolidation as in our patients.

There were only 2 relapses later than 12 months which suggests that one year of this type of treatment might be sufficient for this group of patients.

The behaviour of the patients with mediastinal primaries, who nearly all have T cell Lymphoma (Bernard *et al.*, 1982) was different. These patients continued to suffer relapse throughout the two year period of chemotherapy and subsequently on both the NHL and the intensified T cell protocols. The implications for future treatment of T cell disease are discussed in the companion report (Mott *et al.*, 1984).

It is now well established that the great majority of patients with mediastinal primaries have T cell lymphoma, whereas those with abdominal primaries have B cell lymphoma (Bernard *et al.*, 1982), so the finding that their responses to the same treatment regimen are different is not particularly surprising. A histological corollary of this is the finding in the CCSG trial that different chemotherapy protocols were effective for lymphoblastic versus other histological categories of non-Hodgkin's lymphoma of childhood (Anderson *et al.*, 1983).

The toxicity of this treatment programme was variable as might be anticipated from such a complex schedule. Half of the patients required a 15% or greater reduction in chemotherapy at some stage in their programme, and both survival and FFS were substantially better in that group when compared to those not reported to require dose modifications. The same was true for patients on the more intensive T cell protocol (Mott *et al.*, 1984) and the difference for both trials combined is highly significant. The types of toxicity encountered and their relationship to prognosis will be the subject of a separate report.

The success of this treatment regimen for patients with disseminated disease, particularly those without mediastinal primaries, whether or not they received adjuvant radiation, suggests that exposure of these patients to radiation and the attendant foreseeable hazards of combined radiation/chemotherapy is unnecessary in most instances. The fact that all the patients in the CCSG trial were exposed to both radiation and combination chemotherapy, as were half of the patients in our trial, must give rise to some concern that second malignant neoplasms may occur in the survivors (Donaldson & Kaplan, 1977).

Clear-cut differences in response to treatment are now apparent in a number of subgroups of children with NHL. New treatment strategies based on immunopathological classification, primary site and disease extent are required if results are to be improved further and these will be incorporated in the next generation of trials for these conditions.

We wish to thank the many paediatricians, radiotherapists and surgeons, who have contributed to the management of these patients, and especially our fellow members of the UKCCSG who have worked so hard to make this multidisciplinary, multicentre Trial possible.

We also wish to acknowledge financial support from Lederle Laboratories and the Cancer Research Campaign.

### References

ANDERSON, J.R., WILSON, J.F., JENKIN, D.T. & 8 others (1983). Childhood non-Hodgkin's lymphoma: The results of a randomised therapeutic trial comparing a 4-drug regimen (COMP) with a 10-drug regimen ($LSA_2$-$L_2$). *N. Engl. J. Med.*, **308**, 559.

BERNARD, A., MURPHY, S.B., MELVIN, S. & 4 others (1982). Non-T, non-B lymphomas are rare in children and associated with cutaneous tumor. *Blood*, **59**, 549.

DONALDSON, S.S. & KAPLAN, H.S. (1982). Complications of treatment of Hodgkin's disease in children. *Cancer Treat. Rep.*, **66**, 977.

GOLDMAN, A. (1981). The long term outlook for children treated for non-Hodgkin lymphomas: A report of the Children's Solid Tumour Group. *Br. J. Cancer*, **44**, 872.

HABESHAW, J.A. (1980). Investigation of non-Hodgkin's lymphoma in children by surface phenotyping techniques. In *Non-Hodgkin's Lymphomas in Children*, p. 37. (Ed. Graham-Pole.) Masson, USA.

MOTT, M.G., CHESSELLS, J.M., WILLOUGHBY, M.L. & 4 others (1984). Adjuvant low dose radiation in childhood T cell lymphoma/leukaemia. Report from the United Kingdom Children's Cancer Study Group (UKCCSG). *Br. J. Cancer*, **50**, 000.

MURPHY, S.B. & HUSTU, H.O. (1980). A randomised trial of combined modality therapy of childhood non-Hodgkin's lymphoma. *Cancer*, **45**, 630.

PETO, R., PIKE, M.C., ARMITAGE, P. & 7 others (1977). Design and analysis of randomised trials requiring prolonged observation of each patient. II: Analysis and examples. *Br. J. Cancer*, **35**, 1.

WILSON, J.A., MOTT, M.G. & WILBUR, J.R. (1977). Intensive combination chemotherapy of childhood. Non-Hodgkin's lymphoma. *Amer. Soc. Clin. Oncol.*, **18**, 317.

WOLLNER, N., BURCHENAL, J.H., LEIBERMAN, P.H. & 3 others (1976). Non-Hodgkin's lymphoma in children: a comparative study of two modalities of therapy. *Cancer*, **37**, 123.